# Adapting Patient Priorities Care to Bridge the Digital Divide: A Patient-Centered, Values-Based Approach

**DOI:** 10.1093/geroni/igaf122.2022

**Published:** 2025-12-31

**Authors:** Melissa Hladek, Olivia Rubio, Samantha Curriero, Elisa Letts, Sarah Szanton

**Affiliations:** Johns Hopkins University, Baltimore, Maryland, United States; The Pennsylvania State University, University Park, Pennsylvania, United States; Johns Hopkins University, Baltimore, Maryland, United States; Johns Hopkins University School of Nursing, Baltimore, Maryland, United States; Johns Hopkins University, Baltimore, Maryland, United States

## Abstract

Digital literacy is essential for accessing health information, maintaining social connections, and enhancing quality of life. However, many older adults face barriers to digital engagement particularly those with frailty and from historically marginalized communities, exacerbating healthcare and social disparities. Traditionally, the Patient Priorities Care framework is used in primary care to systematically elicit patient’s values, goals, and healthcare preferences and guide healthcare decision-making and medical treatment options. However, the framework can be applied not only to other healthcare settings, but to broader research and wellness-related fields in order to center the patient’s voice in all aspects of research design and implementation science. To that end, we conducted a randomized control pilot trial enrolling diverse older adults with varying frailty status and digital literacy (N = 20). Over 4 to 6 in-home sessions, interventionists collaborated with participants to identify priorities and co-develop personalized digital strategies and tools based on their goals. Changes in digital literacy and self-efficacy will be reported. Key motivators for technology use included enhancing quality of life, improving health, and connecting with family and friends. Unexpected benefits included engagement with new online communities based on personal interests (e.g., playing chess with global peers). We will also discuss research design-related insights gained from applying the PPC framework in non-traditional ways. Co-designing solutions with participants offers a scalable model to reduce the digital divide and promote equity in aging. Future plans include testing this intervention in a larger trial.

